# Vascular Endothelial Growth Factor Is Associated with the Morphologic and Functional Parameters in Patients with Hypertrophic Cardiomyopathy

**DOI:** 10.1155/2015/762950

**Published:** 2015-12-30

**Authors:** Radek Pudil, Martina Vasatova, Alena Fucikova, Helena Rehulkova, Pavel Rehulka, Vladimir Palicka, Jiri Stulik

**Affiliations:** ^1^1st Department of Internal Medicine-Cardioangiology, Faculty of Medicine in Hradec Kralove, Charles University in Prague, Sokolska 581, 500 05 Hradec Kralove, Czech Republic; ^2^Institute of Clinical Biochemistry and Diagnostics, University Hospital in Hradec Kralove, Czech Republic; ^3^Department of Molecular Pathology and Biology, Faculty of Military Health Sciences, University of Defense, Hradec Kralove, Czech Republic

## Abstract

*Background*. Hypertrophic cardiomyopathy (HCM) is mostly autosomal dominant disease of the myocardium, which is characterized by myocardial hypertrophy. Vascular endothelial growth factor (VEGF) is involved in myocyte function, growth, and survival. The aim of study was to analyze the clinical significance of VEGF in structural and functional changes in patient with HCM.* Methods*. In a group of 21 patients with nonobstructive HCM, we assessed serum VEGF and analyzed its association with morphological and functional parameters. Compared to healthy controls, serum VEGF was increased: 199 (IQR: 120.4–260.8) ng/L versus 20 (IQR: 14.8–37.7) ng/L, *P* < 0.001. VEGF levels were associated with left atrium diameter (*r* = 0.51, *P* = 0.01), left ventricle ejection fraction (*r* = −0.56, *P* = 0.01), fractional shortening (*r* = −0.54, *P* = 0.02), left ventricular mass (*r* = 0.61, *P* = 0.03), LV mass index (*r* = 0.46, *P* = 0.04), vena cava inferior diameter (*r* = 0.65, *P* = 0.01), and peak gradient of tricuspid regurgitation (*r* = 0.46, *P* = 0.03).* Conclusions*. Increased VEGF level is associated with structural and functional parameters in patients with HCM and serves as a potential tool for diagnostic process of these patients.

## 1. Introduction

Hypertrophic cardiomyopathy (HCM) is a heterogeneous myocardial disorder characterized by myocardial hypertrophy with structural and functional abnormalities. The incidence of HCM is approximately 1 in 500 (0.2%) of the general population [1]. The clinical outcome of HCM is diverse, ranging from asymptomatic patients to cardiac arrhythmias, congestive heart failure, and sudden cardiac death. According to the guidelines, patients with HCM require lifelong follow-up to detect changes in symptoms, risk of adverse events, left ventricle outflow tract obstruction (LVOTO), LV function, and cardiac rhythm [1, 2]. The recommended follow-up includes clinical evaluation, 12-lead ECG, and transthoracic echocardiography. The use of laboratory markers seems to be promising for diagnosis and risk stratification in HCM. There is a broad spectrum of biomarkers in peripheral blood, which are potentially useful for diagnosis and risk stratification in patients with HCM. But only cardiac troponins and natriuretic peptides have the most robust data [2]. The previous studies showed that high levels of natriuretic peptides and cardiac troponins are associated with cardiovascular events, heart failure, and death [3]. Systemic evaluation of these parameters revealed an association of these markers with the morphological and functional parameters [4–6]. The association of the myocardial hypertrophy and cardiac troponin and natriuretic peptide levels is not specific for the degree of left ventricle remodeling in HCM. The increase of these parameters is driven probably more by myocardial damage or pressure overload in patients with obstructive type of LV hypertrophy and not by hypertrophy* per se*.

Vascular endothelial growth factor (VEGF) is an endothelial cell-specific mitogen* in vitro* and angiogenic inducer in* in vivo* models. VEGF is secreted as a glycosylated homodimeric protein of 46 kDa that is made up of two 24 kDa subunits linked by disulphide bonds.

The tissue distribution of these VEGF receptors includes vascular smooth muscle cells, osteoblasts, cardiomyocytes, myofibroblasts, neurons, and various tumor cells [7]. It has been shown that VEGF is highly expressed in cardiomyocytes and myofibroblasts, indicating that VEGF family plays an autocrine/paracrine role in the regulation of myocyte and myofibroblast function and growth/survival [8–10]. Vascular endothelial growth factor plays an important role in the process of myocardial hypertrophy. However, the data about the association of VEGF and morphological and functional parameters in patients with hypertrophic cardiomyopathy are missing. The aim of our study was to assess VEGF level in peripheral blood in patients with HCM and to analyze its association with clinical markers of the disease.

## 2. Material and Methods

### 2.1. Study Population

Study population consisted of patients with nonobstructive hypertrophic cardiomyopathy. The diagnosis of hypertrophic cardiomyopathy was based on a history of illness, physical examination, echocardiography, and cardiac catheterization in accordance with European Society of Cardiology recommendations [2]. Based on the results of previous cardiac catheterization, patients with resting or provoked LVOTO were not included in the study. Also, patients with significant concomitant disease, such as pulmonary disease, arterial hypertension, malignancy, autoimmune disorders, neurodegenerative disorders, thyroid disease, or concurrent viral disease, were excluded. VEGF levels were compared to control group of healthy 20 blood donors (40.4 ± 8.5 years) with no evidence of cardiovascular disease according to ECG stress test and echocardiography. The study protocol conformed to the ethical guidelines of the 1975 Declaration of Helsinki and was approved by the ethical committee of our institution. Informed consent was obtained from each patient. Baseline demography and clinical characteristics of the study population are shown in Table 1.

### 2.2. Echocardiography

Echocardiography was performed in agreement with the American Society of Echocardiography and European Association of Cardiovascular Imaging standards evaluating the following parameters: left ventricular end-diastolic (LVEDD) and end-systolic (LVESD) diameters, left atrium diameter (LA), right ventricle diameter (RV), end-diastolic interventricular septum (IVST) and posterior wall thickness (PWT), inferior vena cava diameter (IVC), LV ejection fraction (LV EF), left ventricle fractional shortening (FS), peak gradient of tricuspid regurgitation (Pgrad TR), left ventricle mass (LVM), and left ventricle mass index (LVMI) [11]. Left ventricle outflow tract gradient was evaluated in accordance with European Society of Cardiology guidelines at rest and after provocation by Valsalva maneuver [2].

### 2.3. Assessment of VEGF

Blood samples were obtained from venous catheters, introduced into tube collectors containing no preservatives. Within 1 h, the blood samples were centrifuged for 10 min at 2500 g and the supernatant was removed and kept at −70°C until the assay was performed.

VEGF concentrations were measured using cytokine array for the evidence investigator protein biochip system (Randox Laboratories, UK). Simultaneous quantitative detection of multiple analytes based on sandwich chemiluminescent immunoassay was carried out from a single patient sample. The core technology consists of a solid plate containing discrete test regions with immobilized antibodies specific to different markers. Increased levels of marker in a specimen lead to increased binding of antibody labeled with horseradish peroxidase and thus to an increase in the luminescent signal emitted. The light signals generated from each of the test regions on the biochip are simultaneously detected using a charge coupled device camera. The analytical range of the VEGF assay was 3.24–1000 ng/L. The interassay coefficient of variability (*n* = 20) was, for 146.4 ng/L, 10.8% and, for 456.3 ng/L, 7.4%. Internal quality control measurements were carried out using samples provided by the kit manufacturer.

### 2.4. Statistics

Statistical analysis was performed by MedCalc Software, version 14 (MedCalc Software bvba, Ostend, Belgium). Normally distributed variables are expressed as means ± standard deviation, while nonnormally distributed variables are expressed as median (interquartile range). Categorical variables are presented as percentages. Continuous variables were compared using Student's *t*-test, Mann-Whitney, or Wilcoxon's tests, where appropriate. Linear regression was applied to evaluate the relationship between continuous variables. The degree of association between continuous variables was calculated using Pearson's correlation coefficient. A *P* value <0.05 was considered statistically significant.

## 3. Results

### 3.1. Serum VEGF

**Figure 1:**
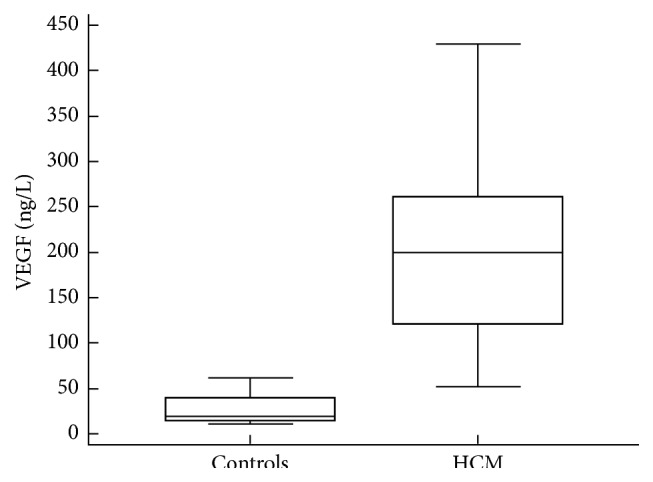
Serum VEGF in patients with hypertrophic cardiomyopathy and controls. HCM, hypertrophic cardiomyopathy.

Serum VEGF was increased in all 21 patients. Compared with controls, HCM patients had significantly increased serum VEGF level 199 (IQR: 120.4–260.8) ng/L versus 20 (IQR: 14.8–37.7) ng/L, *P* < 0.001 (Figure 1). In patients with New York Heart Association (NYHA) functional classes I and II, serum VEGF values were lower compared to patients with NYHA classes III and IV: 146.4 (IQR: 113.6–235.2) ng/L versus 328.1 (IQR: 286.7–406.3) ng/L, *P* < 0.01. In patients with atrial fibrillation, VEGF values were not significantly increased compared to patients with sinus rhythm: 234.1 (IQR: 132.2–307.5) ng/L versus 158.5 (IQR: 121.8–250.1) ng/L, *P* 0.43.

### 3.2. Echocardiography

All patients underwent two-dimensional and Doppler echocardiography.  Table 2 shows the summary information of echocardiographic parameters of patients with hypertrophic cardiomyopathy. The mean of the anteroposterior left atrium dimension was 45.2 ± 5.6 mm, and it exceeded reference values (female: 38 mm, male: 40 mm) in 17 (81%) patients. The mean internal diameter of right ventricle was 26.3 ± 3.2 mm, and it exceeded reference value (31 mm) in 1 (5%) patient. The mean of the internal end-systolic left ventricle dimension was 31.8 ± 8.8 mm, and it exceeded reference values (female: 34.8 mm, male: 39.8 mm) in 2 (10%) patients. The mean of the internal end-diastolic left ventricle dimension was 47 ± 6.3 mm, and it exceeded reference values (female: 52.2 mm, male: 58.4 mm) in 1 (5%) patient. The mean of the interventricular septum thickness was 18.7 ± 2.2 mm, and it exceeded recommended thickness for diagnosis of hypertrophic cardiomyopathy (≥15 mm) in all patients. The mean left ventricle mass was 408.4 ± 11.8 g, and it exceeded reference values for two-dimensional method (female: 150 g, male: 200 g) in all patients. The mean left ventricle mass index was 202.4 ± 53.9 g·m^−2^, and it exceeded reference values for two-dimensional method (female: 88 g·m^−2^, male: 102 g·m^−2^) in all patients. The mean of the left ventricle ejection fraction was 65.4 ± 12.2%, and, only in 1 (5%) patient, the LV ejection fraction was below the reference values (female: 54%, male: 52%). The mean of the left ventricle fractional shortening was 0.32 ± 0.09, and it exceeded the reference values (female: 27–45, male: 25–43) in 3 (15%) patients. The mean peak tricuspid regurgitation gradient was 22.6 ± 11.8 mmHg. The inferior vena cava diameter was 18 ± 3.7 mm.

None of the patients had left ventricle outflow tract obstruction. The peak left ventricle outflow tract gradients were 6 (IQR: 1–7.5) mmHg during at rest measurement and 15 (IQR: 2.2–29) mmHg during Valsalva maneuver. The median values for the peak LVOT pressure gradient were 3 (IQR: 2.2–5) mmHg for at rest measurements and 14 (IQR: 2–22) mmHg during Valsalva maneuver.

### 3.3. Association of VEGF and Morphological and Functional Echocardiographic Parameters

**Figure 2:**
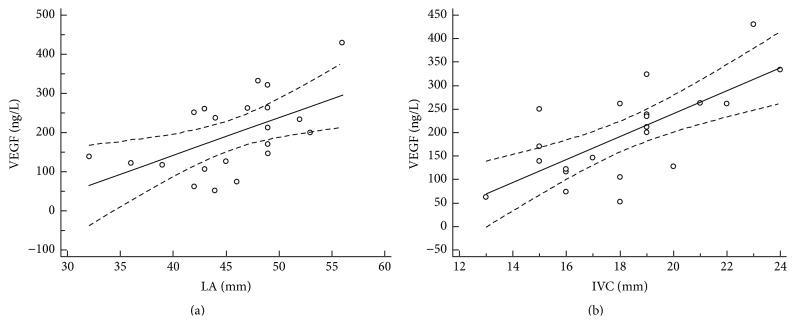
Association of VEGF and left atrium diameter (a) and inferior vena cava diameter (b) in patients with hypertrophic cardiomyopathy. Left atrium (a): *n* = 21, coefficient of determination *R* ^2^: 0.31, regression equation: *y* = −276.4 + 10.4*x*, and significance level: *P* = 0.0082. Inferior vena cava diameter (b): *n* = 21, coefficient of determination *R* ^2^: 0.49, regression equation: *y* = −273.9 + 25.9*x*, and significance level: *P* = 0.0004. LA, left atrium diameter; IVC, inferior vena cava diameter.

**Figure 3:**
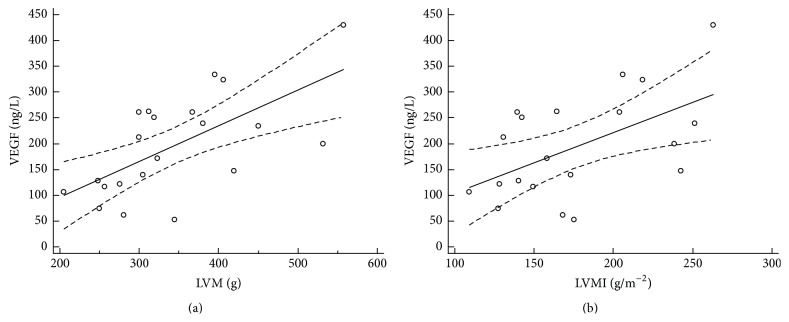
Association of VEGF and left ventricular mass (a) and left ventricular mass index (b) in patients with hypertrophic cardiomyopathy. Left ventricular mass (a): *n* = 21, coefficient of determination *R* ^2^: 0.43, regression equation: *y* = −61.3 + 0.76*x*, and significance level: *P* = 0.0011. Left ventricular mass index (b): *n* = 21, coefficient of determination *R* ^2^: 0.31, regression equation: *y* = −27.5 + 1.3*x*, and significance level: *P* = 0.0115. LVM, left ventricular mass; LVMI, left ventricular mass index.

**Figure 4:**
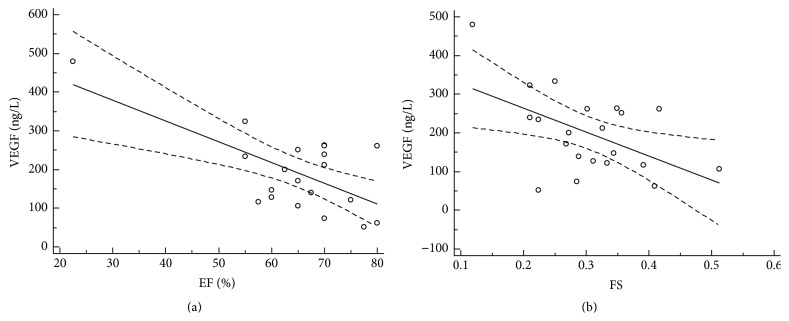
Association of VEGF and left ventricle ejection fraction (a) and left ventricle fractional shortening (b) in patients with hypertrophic cardiomyopathy. Left ventricle ejection fraction (a): *n* = 21, coefficient of determination *R* ^2^: 0.42, regression equation: *y* = 541.3 − 5.4*x*, and significance level: *P* = 0.018. Left ventricle fractional shortening (b): *n* = 21, coefficient of determination *R* ^2^: 0.27, regression equation: *y* = 387.4 − 618.9*x*, and significance level: *P* = 0.0165. LV EF, left ventricle ejection fraction; FS, left ventricular fractional shortening.

**Figure 5:**
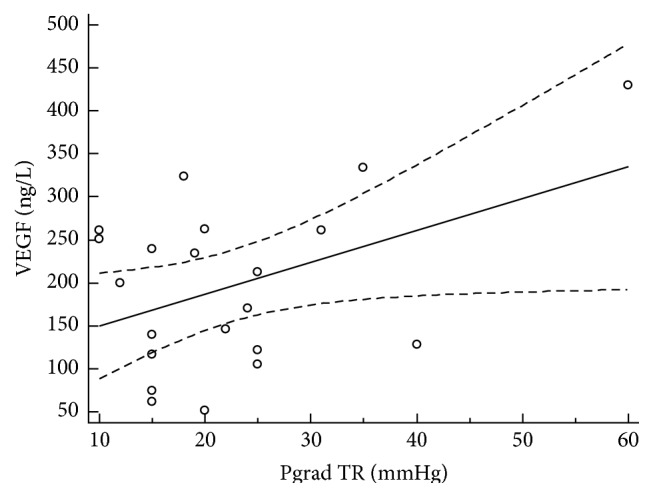
Association of VEGF and peak gradient of tricuspid regurgitation in patients with hypertrophic cardiomyopathy. Peak gradient tricuspid regurgitation: *n* = 21, coefficient of determination *R* ^2^: 0.47, regression equation: *y* = 58.4 + 6.2*x*, and significance level: *P* = 0.005. TR, tricuspid regurgitation.

The analysis of echocardiographic parameters showed association of the morphological and functional parameters with serum VEGF level. Serum levels of increased vascular endothelial growth factor left were significantly associated with left atrium diameter (*r* = 0.51, 95% CI: 0.09–0.77, and *P* = 0.01), left ventricle ejection fraction (*r* = −0.56, 95% CI: −0.80–−0.17, and *P* = 0.01), LV fractional shortening (*r* = −0.54, 95% CI: −0.79–−0.14, and *P* = 0.02), LV mass (*r* = 0.61, 95% CI: 0.24–0.82, and *P* = 0.03), LV mass index (*r* = 0.46, 95% CI: 0.02–0.75, and *P* = 0.04), vena cava inferior diameter (*r* = 0.65, 95% CI: 0.30–0.84, and *P* = 0.01), and peak gradient of tricuspid regurgitation (*r* = 0.46, 95% CI: 0.04–0.74, and *P* = 0.03). Figures 2, 3, 4, and 5 illustrate regression analysis of the morphological and functional echocardiographic parameters.

## 4. Discussion

Hypertrophic cardiomyopathy is an autosomal dominant inherited myocardial disease defined by the presence of increased left ventricular wall thickness that is not solely explained by abnormal loading conditions [2]. The pathophysiology of HCM is complex and consists of multiple interrelated abnormalities, including left ventricle hypertrophy, left ventricle outflow tract obstruction, diastolic dysfunction, mitral regurgitation, myocardial ischemia, and arrhythmias. The natural history of HCM varies from an asymptomatic and benign clinical course to sudden premature death. Therefore, the new markers are searched with the aim for detecting risk patients and improving their prognosis. Until now, there have been published many studies focused on a broad spectrum of biomarkers covering most of the pathophysiological processes described in HCM, for example, myocardial necrosis and wall stress markers, inflammatory markers, markers of endothelial dysfunction, markers of apoptosis, matrix metalloproteinases, platelet function markers, prothrombotic markers, hormones [12–14]. However, no disease specific marker was approved for a routine clinical practice.

In our study, we focused on VEGF and its clinical significance in patients with HCM.

It has been shown that VEGF is an endothelial cell-specific mitogen* in vitro* and an angiogenic inducer in* in vivo* models [15] and plays an important role in myocardial hypertrophy [16]. In our study, we confirmed increased serum VEGF level in patients with nonobstructive HCM when compared to normal population. Furthermore, VEGF levels were significantly associated with the degree of left ventricle hypertrophy (left ventricle mass and its index) in the absence of left ventricle outflow tract obstruction.

The progression of left ventricle hypertrophy leads to development of heart failure and is associated with a poor prognosis [17]. In various studies, plasma natriuretic peptide levels were significantly increased in HCM compared with normal subjects. In HCM patients, both BNP and ANP are significantly higher in the subgroup that shows evidence of obstruction and both correlate positively with left intraventricular pressure gradient. As prognostic factors, plasma levels of NT-pro-BNP and ANP are independent predictors of cardiovascular events in patients with HCM [17–20]. Therefore, we analyzed VEGF level and its relation to clinical and hemodynamic parameters. We observed that the New York Heart functional classes III and IV were characterized by higher VEGF level when compared to NYHA classes I and II. Hemodynamic parameters were evaluated by the use of echocardiography. We found significant negative association of left ventricular functional parameters (ejection fraction and fractional shortening). Also, we have noticed that VEGF is associated with increased pressure in pulmonary artery circulation. The increase of this pressure is passive and it is driven by volume and pressure left ventricle overload. This observation supports left atrium enlargement in the presence of LV hypertrophy. Our results are supported by the previous observations of the pathogenetic role of the VEGF in left ventricle remodeling process in patients with HCM [21–23]. As compared to other markers, VEGF could have a potential to reflect morphological and functional parameters in patients with hypertrophic cardiomyopathy.

## 5. Conclusions

The results of our present study indicate that serum VEGF level in patients with HCM was significantly increased in comparison to control group and was associated with NYHA functional class. The VEGF levels were positively associated with the morphological parameters of HCM (left ventricle mass and its index); functional parameters of the left ventricle showed negative association. Serum VEGF correlated also with peak tricuspid regurgitation gradient (marker of pulmonary circulation pressure), inferior vena cava diameter, and left atrium diameter (markers of volume and pressure overload).

Despite the study limitations (relatively small number of the patients and morphological and hemodynamic parameters were evaluated by echocardiography), serum VEGF has emerged as an interesting and promising parameter useful for risk stratification of patients with hypertrophic cardiomyopathy.

## Figures and Tables

**Table 1:** Patient demographic and clinical characteristics.

	HCM (*n* = 21)
Age (years)	58.4 ± 13.2
Female gender, *n* (%)	6 (28)
Atrial fibrillation, *n* (%)	7 (33)
Creatine level (*μ*mol·L^−1^)	92 ± 14
Diabetes mellitus, *n* (%)	9 (43)
Hyperlipidemia, *n* (%)	14 (66)
Smoking, *n* (%)	7 (33)
NYHA functional class, *n* (%):	
I	8 (38)
II	10 (47)
III-IV	3 (14)
Therapy	
Medication, *n* (%)	
Calcium channel blocker	10 (47)
*β*-blockers	12 (57)
Diuretics	8 (38)
ACE inhibitors/sartans	9 (42)
Implantable devices, *n* (%)	
DDD pacing	5 (24)
ICD/BiV	1 (4)

NYHA, New York Heart Association; ACE, angiotensin converting enzyme; DDD, dual chamber pacing; ICD, implantable cardioverter/defibrillator; and BiV, biventricular pacing.

**Table 2:** Echocardiographic parameters of the study population.

Echocardiography	
LA (mm)	45.2 ± 5.6
RV (mm)	26.3 ± 3.2
IVST (mm)	18.7 ± 2.2
LV ESD (mm)	31.8 ± 8.8
LV EDD (mm)	47.0 ± 6.3
PWT (mm)	13.7 ± 2.0
LV EF (%)	65.4 ± 12.2
LV FS	0.32 ± 0.09
IVC (mm)	18.0 ± 3.7
Pgrad TR (mmHg)	22.6 ± 11.8
LVM (g)	408.4 ± 11.8
LVMI (g·m^−2^)	202.4 ± 53.9

LA, left atrium; RA, right ventricle diameter; IVST, interventricular septal thickness; LV ESD, left ventricular end-systolic diameter; LV EDD, left ventricular end-diastolic diameter; PWT, posterior wall thickness; LV EF, left ventricular ejection fraction; LV FS, left ventricular fractional shortening; IVC, inferior vena cava diameter; Pgrad TR, peak gradient of tricuspid regurgitation; LVM, left ventricle mass; and LVMI, left ventricle mass index.
